# To: Science over language: a plea to consider language bias in scientific publishing

**DOI:** 10.62675/2965-2774.20250027

**Published:** 2025-05-13

**Authors:** Rohan Magoon

**Affiliations:** 1 Atal Bihari Vajpayee Institute of Medical Sciences and Dr. Ram Manohar Lohia Hospital Department of Anaesthesia New Delhi India Department of Anaesthesia, Atal Bihari Vajpayee Institute of Medical Sciences and Dr. Ram Manohar Lohia Hospital - New Delhi, India.

To the Editor

It has indeed been gratifying to have recently read an Editorial by González-Dambrauskas et al.: "Science over language: a plea to consider language bias in scientific publishing", which features at an opportune time to blow an all-important clarion call for Sticking to Science while publishing academic content in scientific journals.^(1)^ In harmony with the noble theme of the index Editorial, i.e., to preserve the health of our research ecosystem, it is believed that the addition of another relevant aspect in this topic would take the discussion further in the right direction.^(1–5)^

Delving deeper into the complex trinity of science, language, and research, it is noteworthy how Lazarus et al. bring the concept of linguistic spin to the fore.^(2)^ Meanwhile, spin, by itself, is characterized by ‘the manipulation of language to potentially mislead readers from the likely truth of the results’; the former research group outlines the utilization of a causal language to have featured as the most prevalent strategy of spin, in a considerable 53% of the abstracts assessed across 128 non-randomized interventional articles.^(2,3)^ Ahead of the matter, which is intertwined between reporting and interpretation, it has something to do with rhetorical additions to manipulate the readers. Interestingly, Lobo, having labeled spin to involve altering the very presentation of facts aided by "disingenuous, deceptive, and manipulative tactics", highlights the larger problem spin may cause when employed in the positive randomized controlled trials providing a base for formulating the clinical care recommendations.^(4)^

Simultaneously, in the specific context of language barriers discussed by González-Dambrauskas et al., SPIN-Prediction Models (SPIN-PM, a consensus framework mapping spin practices) identify the language barriers to facilitate spin in the studies about prediction models, particularly in the background of inexperience and in the absence of requisite guidance.^(1,5)^

Acknowledging the present-day meaningfulness of the Editorial again, it is encouraging to witness serious discussions surrounding the subject of science beyond statistical significance.^(1)^
